# Pain Relief in a Trigeminal Neuralgia Model *via* Optogenetic Inhibition on Trigeminal Ganglion Itself With Flexible Optic Fiber Cannula

**DOI:** 10.3389/fncel.2022.880369

**Published:** 2022-04-28

**Authors:** Elina KC, Jaisan Islam, Soochong Kim, Hyong Kyu Kim, Young Seok Park

**Affiliations:** ^1^Department of Medical Neuroscience, College of Medicine, Chungbuk National University, Cheongju, South Korea; ^2^Department of Veterinary Medicine, College of Veterinary Medicine, Chungbuk National University, Cheongju, South Korea; ^3^Department of Medicine and Microbiology, College of Medicine, Chungbuk National University, Cheongju, South Korea; ^4^Department of Neurosurgery, Chungbuk National University Hospital, Cheongju, South Korea

**Keywords:** neuralgia, trigeminal ganglion, thalamus, pain, optogenetics

## Abstract

The trigeminal ganglion (TG) is the primary site of aberration in trigeminal neuralgia (TN), and hence a crucial site where afferent input can be modulated. Here, we postulated that inhibiting TG *via* optogenetics using flexible optic cannula would diminish brainstem trigeminal nucleus caudalis (TNC) neuronal activity and pain behavior in TN rat model. Infraorbital nerve constriction was employed to induce TN in female Sprague-Dawley rats, while naive and sham rats served as controls. TG-directed microinjections of AAV virus containing either the optogenetic or null vector were delivered to rats in each group. *In vivo* electrophysiological responses were obtained from the ventral posteromedial nucleus (VPm) of the thalamus with simultaneous TG optogenetic stimulation using flexible optic cannula as well the effects on behavioral responses were investigated. Recordings in TN rats revealed a decrease in burst firing activity during yellow laser driven inhibition on TG, as well as considerably improved behavioral responses. In contrast, we noticed persistent hypersensitivity and increased tonic firing with blue laser stimulation which indicates that TG inhibition can synchronize trigeminal pain signal transmission in a TN animal model. The potential of an optogenetic approach in TG itself with flexible optic fiber to directly disrupt the trigeminal pain circuitry delivers fundamental underpinnings toward its prospective as a trigeminal neuralgia management.

## Introduction

Within the pain-transmitting trajectory, the trigeminal ganglion (TG) is a remarkable anatomical and functional structure that integrates nociceptive input from the fair portion of the face in a relatively compact and defined unit (Ajayi et al., 2013). Primary sensory neurons of the trigeminal ganglion mediate the sensation of nociceptive stimuli, which convey noxious information to the brain *via* the medulla oblongata. The TG, which has three major branches, the ophthalmic (V1), maxillary (V2), and mandibular nerves (V3), relays pain perception from the orofacial area (Bista and Imlach, 2019). TG qualifies for a therapeutic influence on the full quarter of the face with limited surgical intervention while preserving the option to explore specific facial areas relevant to recent somatotopic nerve fiber arrangement as a junction among all three trigeminal branches. Orofacial sensory dysfunctions associated with trigeminal nerve injury or orofacial inflammation are probably triggered by altered expression of several molecules in TG neurons, as well as changes in the morphological and physiological aspects of these neurons (Shinoda et al., 2021). Trigeminal neuralgia (TN) is instigated by abnormalities in the afferent neurons of the trigeminal root or ganglion, as per the “ignition theory” (Devor et al., 2002).

Despite significant advances in understanding and treating trigeminal neuralgia, it remains a tricky topic to manage. While many patients respond to first-line therapy for a brief span of time, most therapeutic approaches lose efficacy over time, necessitating the use of substitute remedies (Borroni and Benussi, 2019). Breakthroughs in neuromodulation technology have allowed such refractory cases a remarkable outlook. In the field of electrical neuromodulation, trigeminal ganglion stimulation is a one-of-a-kind therapy option for medically refractory facial pain (van Buyten, 2015). Chronic therapeutic electrostimulation of the trigeminal ganglion has also been seriously considered in patients with persistent trigeminal neuropathic pain (Mehrkens and Steude, 2007).Optogenetics, rather than pharmacological or electrical manipulations, is better for investigating the operational segmentation of neuronal networks that regulate the neural underpinnings of actions because it can provide better specific temporal and spatial control over specific neuronal populations (Mickle and Gereau, 2018).

In addition, changes in TG neurons following peripheral nerve injury are not always equivalent to those in the DRG counterpart of the spinal somatosensory system (Korczeniewska et al., 2020). Alterations in the plasticity of TG neurons elicit allodynia and hyperalgesia, which are predominantly provoked by peripheral nerve injury or orofacial inflammation. It is well documented that the excitability of TG neurons changes in pathological scenarios (Shinoda et al., 2019). Here, we attempted to investigate the role of the trigeminal ganglion during trigeminal neuralgia pathogenesis in an infraorbital nerve chronic constriction model (IONC). Our findings demonstrate that optogenetic stimulation of the trigeminal ganglion seemed to have a substantial effect on addressing atypical symptoms of trigeminal face pain involving the V1-3 area in a TN rodent model.

## Materials and Methods

### Ethics Approval and Consent to Participate

All tests and animal treatments were carried out in accordance with the International Association for the Study of Pain’s ethical guidelines and were authorized by Chungbuk National University’s Institutional Animal Care and Use Committee (IACUC) (CBNUA-1346-20-02). All animal experiments were carried out at Chungbuk National University’s Laboratory Animal Research Center. Every effort was undertaken to minimize the number of rats used and their suffering.

### Animals

Sixty female Sprague Dawley rats aged 8 weeks (weighing 200–250 g on arrival; Koatech, Pyeongtaek, South Korea) were analyzed. Female rats were used since it is widely known that TN-related problems are more common in females than in males (Bangash, 2011). Rats were housed in a temperature- and humidity-controlled conventional area (23°C; 30% humidity) with an equal light-dark period. Clean chow and water were provided *ad libitum*. All the behavioral testing and electrophysiological recording studies reported in this article were carried out by experimenters who were blinded to the treatments during the light hours. Rats were randomly allocated to three groups: Unilateral infraorbital nerve (IONC) constriction (*n* = 35), sham surgery (*n* = 20), and control (*n* = 5).

### Surgical Procedures

For anesthesia, a saline solution containing 15 mg/kg zoletil^®^ (Zoletil50^®^, Virbac Laboratories, Carros, France) and 9 mg/kg xylazine (Rompun^®^, Bayer AG, Leverkusen, Germany) was used intraperitoneally. Chronic constrictive injury of the infraorbital nerve (IONC) rat model of trigeminal neuropathic pain exhibits many features with clinical abnormalities in humans suffering from trigeminal neuralgia or trigeminal neuropathic pain (Deseure and Hans, 2015). We followed the procedure described in other articles (Vos et al., 1994; Kernisant et al., 2008; Deseure and Hans, 2015). Briefly, a small, curved incision was made above the eye region to expose the infraorbital nerve (IoN). The IoN was separated from the surrounding connective tissue and muscle by blunt dissection with Dumont forceps. A 3-0 silk suture was used to create two loose ligations around the right IoN with a gap of 2 mm, and the incision was sealed with silk (3-0). In all rats, the contralateral sides were kept intact. The right IoN was exposed without nerve ligature in sham-operated rats. All surgical procedures were carried out in an aseptic manner.

### Stereotactic Injections

Animals were placed on a stereotaxic platform following IoN constriction, and a viral vector was injected into each group according to the preceding strategy (Whitehead et al., 2003; Elina et al., 2021). Briefly, anesthetized rats were mounted onto a stereotaxic apparatus for precise positioning at the TG (Vit et al., 2009; Tang et al., 2014). A 1-mm-diameter hole was drilled into the skull (3.5 mm anterior from bregma and 3.6 mm lateral to the midline) with a dental drill after the cranial parietal skin was incised to reveal the skull. The tip of the Hamilton syringe was inserted into the right TG (10.4 mm depth from the skull surface). With 10-min intervals, the TN opto/sham opto group received 2 μL of AAV-hSyn-hChR2-mCherry, followed by 2 μL of AAV-hSyn-eNpHR3.0-EYFP (Korea Institute of Science and Technology, Seoul, Republic of Korea) in unilateral TG. The viruses were diluted 1:6 in phosphate-buffered saline (PBS) and delivered for 10 min with a Hamilton syringe and an automated microsyringe pump (KD Scientific Legato^®^ 130 Syringe Pump, Harvard Apparatus, Holliston, MA, United States) with a flow rate of 0.5 μL/min. The needle was held in place at the target for 5 min after viral injection to prevent leakage and then gently retracted during a 5-min period. The concentrations of the ChR2 and NpHR viruses were 1.9 × 10^13^ GC/μL and 2.23 × 10^10^ GC/μL, respectively. We used AAV2−hSyn-EYFP as a control virus at a concentration of 5 × 10^12^ GC/μL. TN null/sham null mice received 4 μL AAV-hsyn-EYFP on TG following the same protocol as above.

To seal the incision, the cut region was sutured. The animals were kept in separate cages and kept in sternal recumbency. The injection site coordinates were determined using the stereotaxic atlas (Paxinos and Watson, 2006). Rats were divided into four groups to examine the influence of optogenetic viruses on neuropathic and sham rats.

### Behavioral Analysis

The behavioral studies mentioned in this article were conducted by researchers who were unaware of the interventions. Before every test, each rat was handled for 30 min and habituated to the experimenter’s hand. The instinctive behaviors in response to mechanical and thermal stimulation were tested a day before (baseline test) and 3, 7, 15, and 21 days after the IoN constriction or sham procedure.

The rat was confined in a Plexiglas arena and recorded. Spontaneous face grooming (SFG) episodes were monitored over a 7-min duration after the rat was placed in the enclosure. An uninterrupted sequence of face grooming acts was described as a face grooming episode (Ding et al., 2017).

After being accustomed to the investigator and the von Frey fibers, rats were evaluated in plexiglass cages until they became unconcerned about their presence in Von Frey filaments test (VF). A set of von Frey filaments (bending forces: 0.4, 1.4, 6, and 15 g) were used to determine mechanical sensitivity (Wu et al., 2016). The filaments were tested in order of escalating force, and the mechanical threshold value was obtained to be a touch stimulus force that elicited a positive (quick and obvious) withdrawal response. Each rat’s and each of the four fibers’ scores were averaged from the three trials.

The mechanical allodynia of the orofacial area is measured using air puff test (APT) in a TN animal model. It is based on the withdrawal of the face in response to continuous air puffs of variable pressures applied to the affected orofacial region (Jeon et al., 2012). After applying 10 consecutive trials of continuous air-puff pressure (4 s length and 10 s intervals) to the trigeminal territory, we looked at withdrawal behavioral responses such as escape from air-puff stimulation or violent actions such as biting. A pneumatic pump module (BH2 system, Harvard Apparatus, United States) controlled the air puff pressure and intervals. Puffs of air were delivered *via* a 26-gauge metal tube (length: 10 cm) held at a 90-degree angle to the skin. The pressure cutoff for air puffs was 40 psi (pounds per square inch). Mechanical allodynia was described as a significant decrease in the air puff threshold compared to baseline values.

After a 10-min habituation period in plexiglass cages, a few drops of 99.7% acetone were applied to the vibrissal pad area, and scratching/aggressive behaviors involving intense head shaking were observed for 2 min in acetone test (AT). Rubbing acts involving areas of the body other than the face were not analyzed (Jeon et al., 2012). The behavioral test was repeated three times at 5-min intervals, with the number of responses summed.

### Acute Electrophysiological Recordings *in vivo*

*In vivo* extracellular recording was conducted on all animal groups 3 weeks after virus injection. The recordings were carried out in a Faraday enclosure in a quiet space with dim lighting. Rats were placed into a stereotaxic apparatus while being anesthetized with a blend of zoletil and xylazine. After adjusting the skull plane to ensure that the bregma and lambda were horizontal, a small craniotomy was performed over the thalamic ventroposterior medial (VPm) area (coordinates from bregma: AP, −3.5 mm; ML, −2.8 mm) of the rat for thalamic recordings (Islam et al., 2021). A quartz-insulated carbon electrode (E1011-20, Carbostar-1, Kation Scientific, Minneapolis, MN, United States) was inserted unilaterally in the VPm thalamus and slowly lowered to the depth (DV, –1.3 mm). The 36-channel headstage and preamplifiers were attached to the electronic interface board (EIB-36, Neuralynx, United States), and the outputs were sent to a Cheetah Acquisition System (Neuralynx, United States). The sampling frequency was set to 30 kHz, and neuronal signal bands were filtered at 0.9–6 kHz in a Digital Lynx SX data-acquisition system (Neuralynx, Bozeman, United States). Neuralynx’s Spikesort 3D software was used to sort the units offline. Waveforms with separable clusters and clear shapes were referred to as single units and exported to NeuroExplorer (version 4, Nex Technologies, Colorado Springs, United States) to obtain tonic firing and burst frequency under different test conditions (Gwak et al., 2010; Liew et al., 2021).

### Optogenetic Modulation Parameters

We used a laser with wavelengths of 473 nm and 589 nm (ADR-700D, Shanghai, China) and a 10-mW output power for optical stimulation. A waveform generator (Keysight 33511B-CFG001, Santa Rosa, CA, United States) powered the laser and allowed the frequency and pulse widths of the square pulses to be adjusted for optical stimulation and inhibition. To avoid undesired heating consequences from constant light, we used intermittent blue laser stimulation for activation. On the other hand, the irradiance can be minimized by an increase in expression density of halorhodopsin in the neurons. On eNpHR3.0 expressing neurons, we employed a minimum irradiance of 0.4 mW/mm^2^ for 100% spiking inhibition under continuous illumination of 593 nm wavelength light. The activity was assessed under light-on and light-off conditions for 10 min each with a 2-min interval period.

### Flexible Optic Fiber Cannula Implantation

Eight rats from each group were anesthetized with zoletil-xylazine (15 mg/kg tiletamine/zolazepam and 9 mg/kg xylazine) and placed into a stereotaxic apparatus. We fastened three anchor screws to the skull for optic cannula implantation and then stereotaxically attached flexible optic fiber cannulae (Goldstone Scientific, Prizmatix, Israel) to unilateral TG (AP: –3.5 mm, ML: 3.6 mm, DV: –10.4 mm). Each cannula is made up of a zirconia ferrule (size-2.5 mm) with a high numerical aperture (0.66) plastic optical fiber (core diameter-250 μm) protruding 1 cm from the ferrule. Both ends of the fiber were optically polished to ensure high-quality optical coupling and durability. Super glue and dental cement (Orthojet Pound Package, Lang Dental, Wheeling, IL, United States) were used to secure the cannula at the same time. The animals were then returned to their home cage for a 7-day recovery period.

### Behavioral Experiments Under Laser Stimulation

Behavioral tests (VF, AT, SFG, AP) were performed with intermittent blue light stimulation for activation and continuous yellow light stimulation for silencing TG neurons for 3 min. The flexible optic cannula implant in TG was illuminated with blue light from a 473-nm laser (BL473T3-100, ADR700D, Shanghai, China) and yellow light from a 589-nm yellow DPSS laser (model: YL589T3-010FC, Shanghai Laser and Optics Century Co., Ltd., Shanghai, China) *via* a monofiber optic patch chord.

### Tissue Processing and Histology

Immediately after optogenetic stimulation, rats were deeply anesthetized with zoletil/xylazine mix and transcardially perfused with PBS followed by 4% paraformaldehyde (PFA). Before embedding, TGs were isolated and fixed in 4% PFA overnight and then submerged in a 30% sucrose solution. We embedded tissues in optimum cutting temperature (OCT, Tissue Tek^®^, Sakura, United States) compound and then cryo-froze them with liquid nitrogen and isopentane. The frozen samples were preserved at –80°C until use and 20 μm TG sections were prepared using a cryostat (Thermo Scientific, Waltham, MA, United States). Evans blue was used to track the site, and cresyl violet staining was used to label TG on sectioned samples. The sections were incubated with DAPI (Vectashield^®^, Vector Laboratories, Inc., Burlingame, CA 94010) and mounted with coverslips. A fluorescence microscope was used to examine viral expression on the slides.

The sections were washed in wash buffer (10X TBS) and blocked-in blocking solution (10% normal goat serum) for 1 h before immunofluorescence labeling of glutamatergic, GABAergic, CGRP, and glial cells. Afterward, the slides were incubated with primary antibodies overnight at 4°C (VGLUT2, Abcam, ab216463; GABA, Sigma–Aldrich, A2025; GFAP, Abcam, ab68428; CGRP, Abcam, ab36001). The sections were washed thoroughly with wash buffer the next day and incubated for 2 h at room temperature with appropriate secondary antibody (Alexa Fluor 488, ab150129, ab150077, Abcam). After another wash in PBS, the slides were mounted with mounting medium (ab104139, Abcam) and coverslips followed by observation under a fluorescence microscope. The images were taken with cellSens Standard software (Olympus Corp., Tokyo, Japan). Immunofluorescence images were merged and quantified with ImageJ software (National Institutes of Health, MD, United States).

### Statistical Analysis

The data are shown as the mean ± standard deviation (SD). G*Power (version 3.1.9.4, Germany) and previous experimental considerations were used to determine the sample sizes. Rats with improper viral expression were not included in the analysis. To compare two groups, Student’s *t*-test or a paired *t*-test was utilized. One- or two-way analysis of variance (ANOVA) followed by Tukey’s *post hoc* test or repeated-measures ANOVA based on the experimental terms were used to compare three or more groups. To compare the quantification of immunofluorescence images, we used unpaired *t*-test. In all cases, the significance threshold was set at *P* < 0.05. GraphPad Prism (version 8.4.2, Inc., San Diego, CA, United States) was used to conduct the statistical analyses.

## Results

### Infraorbital Nerve Induces Hypersensitivity and Allodynic Behaviors

**FIGURE 1:**
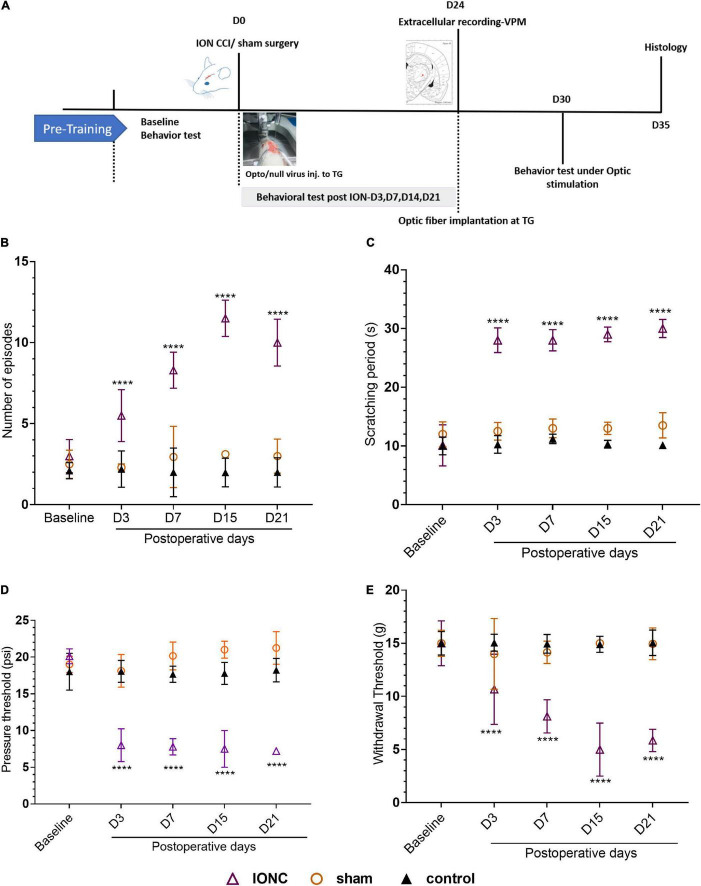
Study design and orofacial pain behavior test. **(A)** Experimental design shown in days. **(B)** Incline in number of episodes in face grooming test following IONC. **(C)** Increased scratching period (in seconds) in acetone test was notedin IONC. **(D)** Decreased air pressure threshold (in psi) in ipsilateral orofacial area following IONCin air puff test. **(E)** Decreased mechanical face withdrawal threshold (in grams) following IONC. In **(B–E)**, data are presented as the mean ± SD. ****P < 0.0001 (two-way ANOVA) compared to sham and control group.

Chronic constriction of the infraorbital nerve was used to generate the TN rat model, which emulates clinical TG. The experimental design is presented in Figure 1A. Three days after IONC, mechanical allodynia was detected in the ipsilateral vibrissa pad. Asymmetric face grooming episodes were also more common in TN rats than in sham and control rats (10 ± 1.44 vs. 3 ± 1.05 vs. 2 ± 0.90, *p* < 0.0001, Figure 1B). All groups were tested in an APT and AT to examine the pressure and thermal threshold simultaneously. In the AT, the facial scratching period significantly increased from 10.084 ± 3.50 s to 30 ± 1.55 s over the 21-day period in the IONC group [two-way ANOVA, *F*_(4,115)_ = 119.3, *p* < 0.0001, Figure 1C]. The increase in mechanical sensitivity was reflected in the significant decrease in the mechanical response threshold in the ipsilateral vibrissa pad in the VF test after IONC (15 ± 2.1 g to 5.86 ± 1.05 g, Figure 1D). The response to mechanical stimulation of the contralateral IoN territories in these same rats was not distinctly significant; however, an increase in sensitivity was observed. In addition, a gradual decrease in the APT was also noted on the ipsilateral side [20.10 ± 1.01 psi to 7.20 ± 0.12 psi, two-way ANOVA, *F*_(4,115)_ = 55.46, *P* < 0.0001, Figure 1E] at 21 days postsurgery. These data demonstrate that TN was successfully induced with infraorbital nerve ligation and caused allodynia and hypersensitive behaviors.

### Optogenetic Modulation of Trigeminal Ganglion Neurons Alters Ventroposterior Medial Thalamic Discharge

**FIGURE 2:**
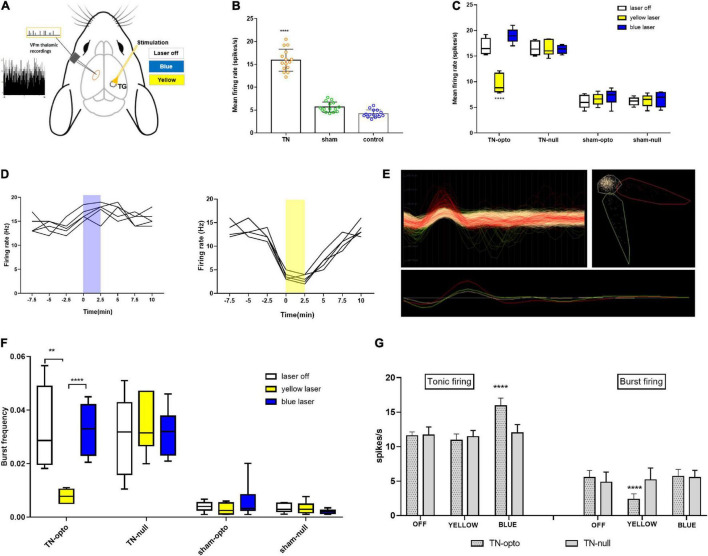
In vivo extracellular recording from the VPm thalamus in response to TG optogenetic modulation. **(A)** Schematic diagram for in vivo recording. **(B)** Mean firing rate comparison between the groups (in spikes per second). **(C)** Mean firing rate from the VPm thalamus between the TN (opto and null) and sham (opto and null) groups inresponse to different stimulation states (laser off, yellow laser, and blue laser) in the TG. **(D)** Representative firing rate (in Hertz) during blue and yellow laser stimulation in the TN-opto group. **(E)** Representative cluster sorting from different waveforms in spike sorter software. **(F)** Burstfrequency comparison between TN (opto and null) and sham (opto and null) groups in response to different stimulation states (laser off, yellow laser, and blue laser) in TG. **(G)** Tonic and burst firing (spikes per second) comparisons between the TN-opto and TN-null groups in response to different stimulation states (laser off, yellow laser, and blue laser) in TG. Data are presented as the mean *±* SD. **P *<* 0.01, ****P *<* 0.0001 compared to other groups (two-wayANOVA).

To address whether TN altered the excitability of thalamic neurons, the electrophysiological characteristics were examined in the VPm thalamus using single-unit extracellular recordings at the fourth week after ION under different conditions (Figure 2A). We identified that TN rats had a significantly higher mean firing rate (15.91 ± 2.414 spikes/s) than sham (5.466 ± 1.109 spikes/s) and control (4.213 ± 0.8314 spikes/s) rats (Figure 2B). Blue light activation of TG neurons significantly increased tonic firing (laser off: 16.853 ± 1.549; laser on: 18.977 ± 1.37 spikes/s) of the VPm thalamus in TN-opto rats, but no noticeable change was observed in burst firing (Figures 2C,D,F,G). In contrast, we found decreased bursting activity during inhibition of TG neurons in the TN-opto group, but no significant changes were observed in the other groups (Figures 2C,D,F,G). Figure 2E is an exemplary cluster sorted from several waveforms. We noted that there was little change in the tonic firing pattern, but the mean firing rate was reduced with the optogenetic inhibition of TG neurons in TN-opto animals. This implies that NpHR triggers a key influence on bursting activity and that this hyperpolarization marks an overall decline in thalamic discharge in neuropathic rats.

### Optogenetic Activation of Trigeminal Ganglion Neurons Caused Persistent Trigeminal Neuralgia-Induced Hypersensitivity

**FIGURE 3:**
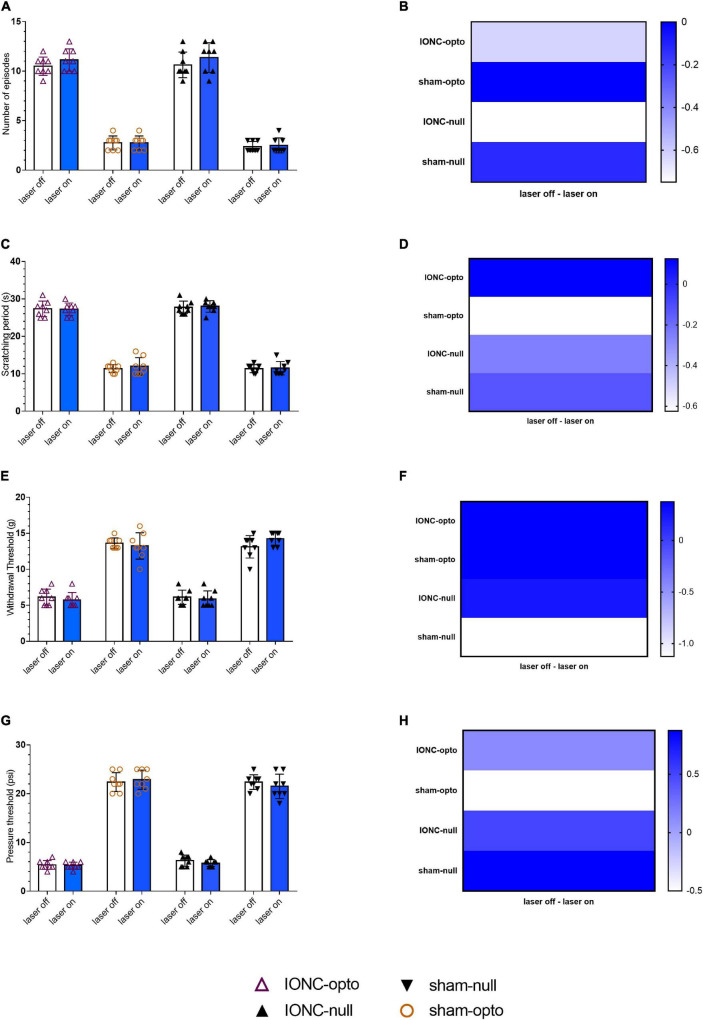
Persistent hypersensitivity by TG optogenetic activation. **(A)** Sustained hypersensitive behavioral activity in response to blue laser stimulation in the face grooming test. **(B)** Heatmap for face grooming test. **(C,D)** Cold acetone test response to blue laser stimulation in TG neurons.**(E,F)** Persistent decreased air pressure threshold in the air puff test with blue laser stimulation in the IONC opto groups. **(G,H)** Mechanical threshold result in response to TG activation with a blue laser in the von Frey filament test. Data are presented as the mean ± SD. No significant differences were noted in two-way ANOVA.

We examined pain behavior tests in the blue laser-on and blue laser-off conditions in all four groups of animals. There was no significant difference between the two states when analyzing the effects of the blue laser in TN and sham rats. During optogenetic activation of the trigeminal ganglion, a behavioral study demonstrated that TN rats receiving optogenetic virus displayed sustained hypersensitive responses, as evidenced by poor mechanical and pressure thresholds in the von Frey and air puff tests, respectively. Additionally, similar results were noted in spontaneous grooming and acetone tests with constant scratching behaviors. IONC rats injected with optogenetic virus showed an amplified number of episodes in blue light off (10.50 ± 0.926) and on (11.125 ± 1.126) settings (Figures 3A,B). They also had no differences in the scratching period (laser off: 27.375 ± 2.066 s; laser on: 27.25 ± 1.669 s, Figures 3C,D). The whisker pad withdrawal threshold in the von Frey test was 6.12 ± 1.26 g in the laser off state, which was like that in the laser on state (5.75 ± 1.035 g, Figures 3E,F). The pressure threshold in the air puff test was also similar in the blue laser off vs. on states (5.375 ± 0.916 vs. 5.25 ± 0.707 psi, Figures 3G,H). We also observed trivial variations in the null virus-injected and sham groups.

### Inhibition of Trigeminal Ganglion Neurons Through NpHR Activation Produces Antinociceptive Effects

**FIGURE 4:**
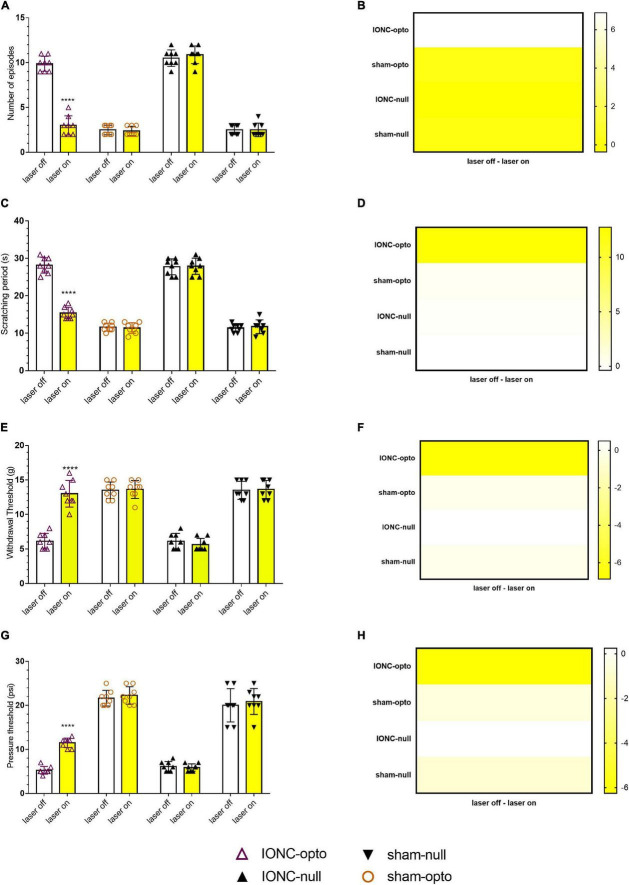
Hypersensitivity induced by IONC improved with TG neuron optogenetic inhibition. **(A)** Decreased number of episodes noted in response to yellow laser stimulation in the face grooming test. **(B)** Heatmap for face grooming test. **(C,D)** Cold acetone test response in TG neurons with yellow laser stimulation. **(E,F)** In the IONC opto groups, yellow laser stimulation increased the air pressure threshold in the air puff test. **(G,H)** In the von Frey filament test, an improved mechanical threshold was revealed in response to TG neuron inactivation with a yellow laser. Data are presented as the mean ± SD. ****P < 0.0001 compared to other group (two-way ANOVA).

Spontaneous grooming (laser off: 9.875 ± 0.835; laser on: 3 ± 1.069, Figures 4A,B) and cold allodynic (laser off: 28.125 ± 2.10; laser on: 11.375 ± 1.506, Figures 4C,D) behaviors were improved during stimulation with yellow light. The behavioral test showed that, following optogenetic inhibition, TN rats receiving optogenetic virus exhibited antinociceptive behaviors, marked by increased mechanical (laser off: 6.125 ± 1.126 g; laser on: 13 ± 1.927 g, Figures 4E,F) and pressure (laser off: 5.25 ± 0.886 psi; laser on: 11.50 ± 1.069 psi, Figures 4G,H) thresholds in the VF and APT, respectively. There was no difference between sham rats receiving optogenetic virus and null virus, suggesting that optogenetic modulation is triggered only in neuropathic pain conditions.

### Optogenetic Modulation Alters CGRP Expression in Trigeminal Ganglion Neurons

**FIGURE 5:**
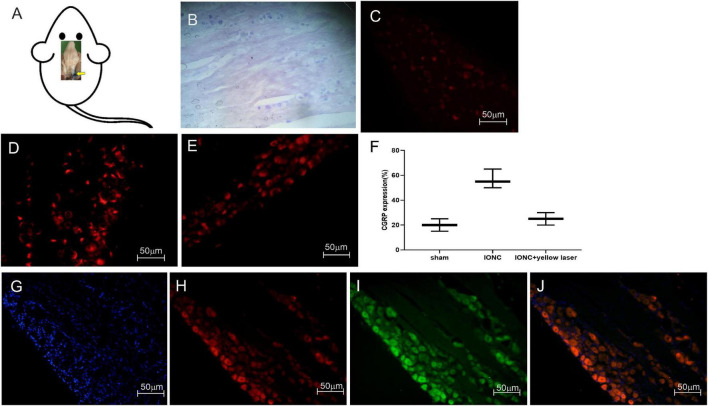
Stereotaxic injection tracking, optogenetic virus colocalization and CGRP expression in TG. **(A)** Tracking of the target site (trigeminal ganglion) with Evans blue dye after stereotaxic injection following sacrifice. **(B)** Cresyl violet staining of the trigeminal ganglion. When compared to a sham control **(C)**, immunofluorescence images demonstrate a significant increase in CGRP expression afterIONC **(D)**. **(E)** Decreased CGRP expression following optogenetic inhibition in TG neurons with yellow laser stimulation. **(F)** Quantified CGRP expression of immunofluorescent images in different conditions (*n* = 3 in each group). **(G)** DAPI, **(H)** mCherry, **(I)** EYFP, **(J)** merged images for colocalization of optogenetic virus (NpHR and ChR2) expression in TG neurons. Sample size in each group = 3. Scale = 50 μm.

Following sacrifice, we employed Evans blue dye to track the injected site (Figure 5A). The normal anatomical structure was stained with cresyl violet and is depicted in Figure 5B. CGRP is upregulated in neuropathic pain, and it has been linked to the development of neurogenic inflammation. We noticed that CGRP expression was higher in pain models (56.67 ± 6.25%) than in sham models (20 ± 4.08%) (Figures 5C,D). After optogenetic inhibition, we observed that CGRP expression was significantly reduced (25 ± 4.08%) in TG neurons (Figures 5E,F). The statistical significant differences (*p*-value = 0.0006^***^) between groups were observed in ordinary one way ANOVA test. Quantified CGRP expression (in percentage) in different groups (*n* = 3 from each group) is shown in Figure 5F.

### Colocalization of Optogenetic Viruses in the Trigeminal Ganglion

To enable specific activation of TG neurons, the blue light-activated cation channel ChR2 was expressed under the control of the hSyn promoter. In AAV-ChR2 animals (*n* = 3), ChR2 was fused to mCherry, enabling visualization of the fusion protein in TG neurons (Figures 5G,H,J). The yellow light-activated anion channel halorhodopsin (NpHR) was expressed under the control of the hSyn promoter to enable targeted inhibition of TG neurons. Halorhodopsin was fused to EYFP in AAV-NpHR animals (*n* = 3), allowing illumination of the fusion protein in TG neurons (Figures 5G,I,J).

### Alterations in VGLUT2, GABA, and GFAP Expression Following Trigeminal Ganglion Inhibition

We observed increased VGLUT2 expression following injury (62.33 *±* 5.24%), but optogenetic inhibition of the TG altered (15 *±* 4.08%) this expression (Figure*s* 6A,B,G). In contrast, we observed that GABA expression was diminished (21.67 *±* 6.25% 0 in IONC animals and was elevated (71 *±* 2.94%) when treated with a yellow laser (Figure*s* 6C,D,H). This is important evidence for decreased GABA inhibition in neuropathic pain states. With optogenetic inhibition in the TG, GABA activity is enhanced and provides an ameliorating effect. We also identified that SGCs in the TG were activated, as shown by enhanced GFAP expression (53.67 *±* 7.84%). Optogenetic inhibition in TG regulated SGC (12.33 *±* 2.05%) activity as well (Figure*s* 6E,F,I). Nevertheless, we cannot preclude the possibility that GFAP-positive cells are not the primary SGCs with enhanced expression but rather a unique subset of glial cells that upregulate GFAP in response to nerve injury (Vit et al., 2006). Quantified data of VGLUT2, GABA, and GFAP expression in immunofluorescence analysis are presented in Table 1.

**TABLE 1:** Quantification of VGLUT2, GABA, and GFAP expression in immunofluorescence analysis.

	Mean (%)	SD	*p*-value (Unpaired *t*-test)
**VGLUT2**			
IONC (*n* = 3)	62.33	5.24	0.0005***
IONC + laser (*n* = 3)	15	0.8	
**GABA**			
IONC (*n* = 3)	21.67	6.25	0.0005***
IONC + laser (*n* = 3)	71	2.94	
**GFAP**			
IONC (*n* = 3)	53.67	7.84	0.0020**
IONC + laser (*n* = 3)	12.33	2.05	

*IONC, infraorbital nerve constriction models; IONC + laser, IONC models modulated with yellow laser in trigeminal ganglion; VGLUT2, Vesicular-glutamate transporter 2; GABA, gamma-Aminobutyric acid; GFAP, Glial fibrillary acidic protein.*

***p < 0.001 and **p < 0.01 considered significant (student’s unpaired t-test).

## Discussion

This study explored how inhibiting trigeminal ganglion neurons *via* NpHR suppressed orofacial nociceptive responses in neuropathic animals with constricted infraorbital nerve using optogenetic techniques. NpHR-mediated TG inhibition led to a decreased response of brainstem trigeminal neurons and behavioral hypersensitivity. The TG is distinctive among the somatosensory ganglia in terms of topography, structure, composition, and presumably some functionality of its cellular components (Messlinger and Russo, 2019). The main sensory neurons of the head and face transmit pain through the TG. As a result, TG-linked components have been a widely held concern in TN research (Liu et al., 2020). All such observations point to the importance of the TG in the circuitry of trigeminal neuropathic pain.

The TG has been termed a “central hub” in the trigeminovascular transmitting pathway, as it contains the soma of the peripheral nerves able to activate superior-order sensory neurons inhabiting the TCC, which in turn progress the signal to the thalamus and finally the cortex (Edvinsson et al., 2020). In the ascending pain circuits throughout the thalamus, somatosensory cortex, and limbic system, elevated neuronal excitability and reduced inhibition are often noted, which may contribute to heightened orofacial neuropathic pain following trigeminal nerve injury (Gambeta et al., 2020). Trigeminal thalamic ventral posteromedial nucleus (VPm) neurons display greater spontaneous activity, wider receptive field size, low activation threshold, and hence heightened excitability following trigeminal nerve injury or inflammation (Aguilar and Castro-Alamancos, 2005). The TG is primarily comprised of pseudounipolar primary afferent neurons and glial cells with cell bodies surrounded by a single layer of SGCs (Nagakura et al., 2021). Neuronal activation occurs, and various molecules are up- or downregulated in the TG in response to trigeminal nerve injury, which is apparent in the peripheral nervous system (Suzuki et al., 2013). Similarly, the present work demonstrated increased thalamic activity following injury.

Crosstalk in the TG modulates neuronal signaling, which leads to inflammatory pain and is a possible therapeutic target for allodynia/hyperalgesia prevention (Goto et al., 2016). Neuropeptides and potassium channels are downregulated in TG neurons following nerve transection, but sodium channels are upregulated and accumulate at the stump end of nerve resection (Liao et al., 2020). After injury, high-frequency action potentials are evoked in primary afferent neurons that innervate orofacial areas. TG neurons become excitable and hypersensitive for a considerable time post trigeminal nerve injury. Satellite cells are activated within the TG in conjunction with TG neuron hyperactivity (Takeda et al., 2016; Okutsu et al., 2021). The trigeminal brain stem nuclei carry heightened nociceptive information from the TG to the upper CNS regions, resulting in intense pain in the orofacial region. The cell bodies of most trigeminal afferents are positioned in the TG (Iwata and Shinoda, 2019). Our results corroborate previous reports wherein modulation of TG neuronal activities regulated thalamic discharge related to neuropathic pain (William et al., 2016). We found that inhibition of TG neuronal activity decreased the mean firing rate of the VPm thalamus.

A cascade of action potentials is established in small-diameter primary afferent neurons after painful stimuli are presented to the orofacial region, and they are relayed to small TG neurons (Costa and de Almeida Leite, 2015). Changes in the excitability of TG neurons are generally triggered by peripheral nerve injury resulting in allodynia and hyperalgesia (Choi et al., 2016). Signs of altered glutamatergic neurotransmission in the sensory ganglia have been reported in numerous pain models based on nerve injury (Fernández-Montoya et al., 2018). Increased GABA levels in the TG have been shown to impact neuronal excitability and modify sensory transmission. Consistent with a previous report, the present results clearly indicate that the hypersensitivity that is usually associated with neuropathy after trigeminal nerve injury was markedly due to glutamatergic neuron hyperactivity and decreased GABA inhibition activity in the TG. In line with earlier research (Kustermans et al., 2017; Ranjbar Ekbatan and Cairns, 2021), we identified that modulating the TG assists in ameliorating hypersensitivity.

Peripheral injury activates intracellular signaling transduction pathways in nociceptor terminals, resulting in a lower activation threshold and higher action potential firing frequency. CGRP is a neuropeptide found in the central and peripheral nervous systems, as well as sensory neural ganglia, that functions as a vasodilator. The TG potentially releases the majority of CGRP, and DRG neuron cell bodies convey them rapidly through the axon to the synapses, sending signals. CGRP is elevated in the TG of TN rats, implying that it likely plays a significant role in neuralgic pain transmission (He et al., 2020). Likewise, we found increased CGRP expression in the TG following IONC, which was altered following optogenetic inhibition with a yellow laser. Decreased CGRP immunoreactivity might be responsible for attenuating behavioral hypersensitivity (Maegawa et al., 2021) following TG inhibition.

It has also been reported previously that VGLUT2-mediated glutamate transmission *via* nociceptive afferent central terminals may be amplified following injury, contributing to hyperalgesia48 (Laursen et al., 2014). Multiple components of the plasticity that occurs in the CNS post peripheral nerve injury have indeed been associated with GABAergic inhibition (Vit et al., 2009). Increased GABA levels in the TG have been shown to impact neuronal excitability and modify sensory transmission (Ranjbar Ekbatan and Cairns, 2021). In line with this hypothesis, we demonstrated that NpHR-mediated inhibition in TG might contribute to modulating GABAergic activity, resulting in a pain-relieving impact. Ectopic orofacial hyperalgesia is caused by the activation of SGCs surrounding the neuronal soma of injured nerve branches extending into the TG region, where the soma of uninjured nerve branches localizes, resulting in ectopic orofacial hyperalgesia (Shin et al., 2020). Taken together, utilizing an optogenetic strategy to directly manipulate TG activity could govern the altered components in pain states and result in antinociception (Figure 7).

**FIGURE 6:**
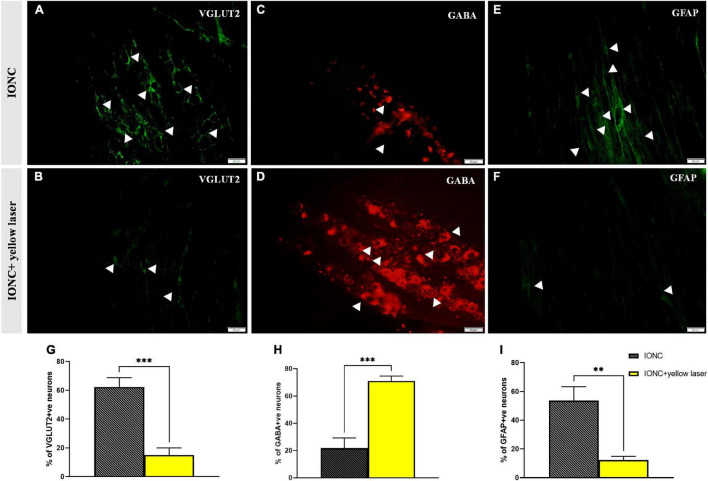
VGLUT2, GABA and GFAP immunofluorescence expression in the trigeminal ganglion. **(A)** Increased VGLUT2 expression in IONC; shown with white arrows. **(B)** NpHR-mediated TG inhibition reduced VGLUT2 expression in the trigeminal ganglion, depicted with white arrows. **(C)** Reduced GABA expression in IONC rats, shown with white arrows. **(D)** Yellow laser stimulation in the TG resulted in increased GABA expression, as shown by white arrowheads. **(E)** Increased GFAP expression in IONCs, as shown by white arrows. **(F)** GFAP expression following yellow laser stimulation in the trigeminal ganglion; shown with white arrows. Scale = 50 μm. **(G)** Quantification of VGLUT2 expression (expressed in percentage) in laser on and off conditions of injured animals. Significant alteration was observed in unpaired *t*-test (^***^p = 0.0005, *n* = 3). **(H)** GABA expression (given as a percentage) in injured animals under laser on and off circumstances. Significant alteration was observed in unpaired *t*-test (^***^p = 0.0005, *n* = 3). **(I)** Quantification of GFAP expression (given in percentage) in laser on and off conditions of injured animals. Significant alteration was observed in unpaired *t*-test (^**^p = 0.0020, *n* = 3). Data are presented as the mean ± SD.

**FIGURE 7:**
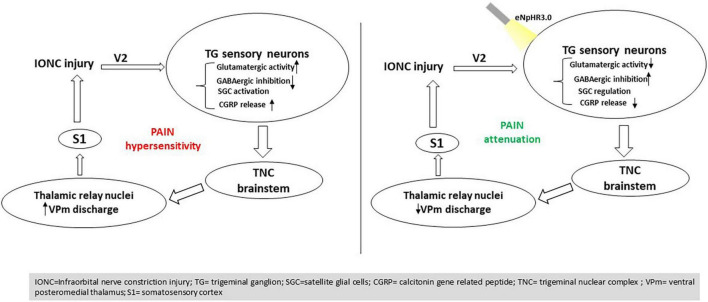
Summary of the findings. Optogenetic inhibition in the trigeminal ganglion influences trigeminal brainstem and thalamic relay nuclei directing to the S1 cortex, resulting in pain mitigation induced by nerve injury.

Remarkably, there were some limitations in the present study that needed to be considered. Even though all animals adequately attended postsurgery, pain chronicity might be influenced by postoperative stress. In addition, we monitored neural recordings in an anesthetized state and thus might have impacted thalamic discharge; however, we employed von Frey filaments to trigger whisker pads to avoid such effects. We performed both spontaneous and evoked electrophysiological recordings. The anesthetic circumstances were found to have a significant impact on spontaneous recordings. Thus, the electrophysiological data that we mentioned in our study are evoked responses to avoid this discrepancy. Here, we mainly focused on stereotactic optogenetic modulation in the TG, but further comprehensive aspects regarding specific neurons have yet to be studied but will be examined in future research.

In trigeminal neuropathy, the direct application of optogenetic methods in TG modulation is still intending to be addressed. The target specificity of such strategies offer good prospects and provide initial evidence. The present study suggests that halorhodopsin-mediated inhibition in the trigeminal ganglion in a rat model result in thalamic activity manipulation as well as GABA disinhibition, which might be significant in therapeutic interventions.

## Data Availability Statement

The raw data supporting the conclusions of this article will be made available by the authors, without undue reservation.

## Ethics Statement

The animal study was reviewed and approved by the Chungbuk National University’s Institutional Animal Care and Use Committee.

## Author Contributions

EK designed the study, carried out the majority of the experiments and statistical analyses, and drafted the manuscript. JI participated in behavioral testing and data acquisition. YP participated in the study design and experimental supervision. SK and HK revised the manuscript and provided valuable suggestions. All authors read and approved the final manuscript.

## Conflict of Interest

The authors declare that the research was conducted in the absence of any commercial or financial relationships that could be construed as a potential conflict of interest.

## Publisher’s Note

All claims expressed in this article are solely those of the authors and do not necessarily represent those of their affiliated organizations, or those of the publisher, the editors and the reviewers. Any product that may be evaluated in this article, or claim that may be made by its manufacturer, is not guaranteed or endorsed by the publisher.
